# Suppression of *Propionibacterium acnes*-Induced Dermatitis by a Traditional Japanese Medicine, Jumihaidokuto, Modifying Macrophage Functions

**DOI:** 10.1155/2015/439258

**Published:** 2015-10-01

**Authors:** Kyoji Sekiguchi, Junichi Koseki, Kazuaki Tsuchiya, Yosuke Matsubara, Seiichi Iizuka, Sachiko Imamura, Takashi Matsumoto, Junko Watanabe, Atsushi Kaneko, Setsuya Aiba, Kenshi Yamasaki

**Affiliations:** ^1^Tsumura Research Laboratories, Tsumura & Co., 3586 Yoshiwara, Ami-machi, Inashiki-gun, Ibaraki 300-1192, Japan; ^2^Department of Dermatology, Graduate School of Medicine, Tohoku University, 1-1 Seiryo-machi, Aoba-ku, Sendai, Miyagi 980-8574, Japan

## Abstract

*Purpose*. Macrophages serve as sweepers of microbes and inflammation-derived wastes and regulators of inflammation. Some traditional Japanese medicines are reported to have adjuvant effects by modifying macrophages. Our aim was to characterize the actions of jumihaidokuto (JHT) for treatment of skin inflammations including acne vulgaris, in which *Propionibacterium acnes* has pathogenic roles. *Methods*. Dermatitis was induced in rat ears by intradermal injection of *P. acnes*. JHT or prednisolone (PDN) was given orally, and ear thickness and histology were evaluated. The effects of constituents and metabolites of JHT on monocytes were tested by cell-based assays using the human monocytic THP-1 cell. *Results*. JHT and PDN suppressed the ear thickness induced by *P. acnes* injection. Histological examinations revealed that JHT, but not PDN, promoted macrophage accumulation at 24 h after the injection. PDN suppressed the macrophage chemokine MCP-1 in the inflamed ears, while JHT did not affect it. The JHT constituents liquiritigenin and isoliquiritin increased expression of CD86 (type-1 macrophage marker) and CD192 (MCP-1 receptor) and enhanced phagocytosis by THP-1. *Conclusions*. JHT suppressed dermatitis, probably by enhancing type-1 macrophage functions, with an action different from PDN. JHT may be a beneficial drug in treatment of skin inflammation induced by *P. acnes*.

## 1. Introduction

Inflammation is the living body's response to both exogenous and endogenous stimuli in order to exclude harmful stimuli and promote repair of injured tissue. The inflammatory spiral has recently been studied in order to understand the pattern recognition receptors and damage-associated molecular patterns involved in sterile inflammation [1, 2]. Neutrophils have high ability to kill foreign microbes but are remarkably short-lived and die at sites of inflammation. Excessive release of reactive oxygen species, reactive nitrogen species, and proteases from dead neutrophils can damage surrounding healthy cells and lead to formation of abscesses and pustules [3, 4]. Macrophages help regulate sterile inflammation. The targets of phagocytosis by macrophages are not only microbes but also inflammation-derived wastes, including dead neutrophils. Type-1 macrophages expressing CD86 are highly phagocytic and play critical roles in infections and cancers to protect the host [4, 5]. Type-2 macrophages directly and indirectly attenuate the inflammatory spiral, followed by promotion of wound healing [6]. Thus, activation of macrophages is critical in sterile inflammation, the inflammatory response to cell and tissue damage, and the shift from acute to chronic inflammation.

Jumihaidokuto (JHT), a pharmaceutical-grade traditional Japanese medicine (Kampo), has been used widely for treatment of skin symptoms such as reddening, swelling, sharp pain, and a sense of burning, as well as skin diseases including acute and/or purulent skin diseases, urticaria, eczema, and athlete's foot. It is reported that JHT is effective in patients with inflammatory acne [7]. Nose et al. found that JHT inhibited hapten-induced inflammation in a mouse model of allergic dermatitis [8]. Akamatsu et al. demonstrated adjuvant effects of JHT in cell-based assays using human neutrophils and reported that JHT increased cell chemotaxis, phagocytosis, and the intracellular concentration of calcium in neutrophils [9]. However, how JHT exerts its pharmacological effects* in vivo* and which constituents of JHT are active in its efficacy still remain to be unknown.

Some Kampo medicines exert adjuvant effects through modification of macrophages [10–12]. Therefore, we hypothesized that JHT may exhibit anti-inflammatory functions by modifying the association between neutrophils and macrophages. In this study, we utilized a rat model of acute dermatitis induced by intradermal injection of* Propionibacterium acnes*, a Gram-positive anaerobic microbe, considering that JHT is prescribed clinically for treatment of a variety of acute and/or purulent skin diseases and is effective in acne patients [7]. We first examined whether JHT attenuated inflammation in* P*.* acnes*-induced dermatitis, in which both neutrophils and macrophages are involved in the pathological process. We also identified the active compounds of JHT constituents and metabolites reported to be absorbed into the systemic circulation. The results of our study suggested JHT and its constituents and metabolites modified the* P*.* acnes*-induced dermatitis by augmenting macrophage functions.

## 2. Materials and Methods

### 2.1. JHT and Test Compounds

JHT was supplied by Tsumura & Co. (Tokyo, Japan) in the form of a powdered extract. It was obtained by spray-drying a hot water extract mixture of the following ten crude components in the ratios provided in parentheses: Platycodi radix (14.3), Bupleuri radix (14.3), Cnidii rhizoma (14.3), Poria sclerotium (14.3), Quercus cortex (14.3), Araliae Cardatae rhizoma (7.1), Saposhnikoviae radix (7.1), Glycyrrhizae radix (4.8), Schizonepetae spica (4.8), and Zingiberis rhizoma (4.8).

Four constituents of JHT and two metabolites converted in a living animal body were selected (see supplementary Figure S1 in the Supplementary Material available online at http://dx.doi.org/10.1155/2015/439258). Liquiritin, liquiritigenin, isoliquiritin, and 18*β*-glycyrrhetinic acid (main metabolite of glycyrrhizin), which are constituent or metabolite of Glycyrrhizae radix, 4-*O*-methylgallic acid (the main metabolite of gallic acid), which is found in Quercus cortex, and cimifugin, which is a constituent of Saposhnikoviae radix, were supplied to this study. Liquiritin and 4-*O*-methylgallic acid were purchased from Wako Pure Chemical Industries (Osaka, Japan), and the other four compounds with purities high enough to be evaluated in biological tests were obtained from the Kampo Formulations Development Center, Tsumura & Co. These six compounds are absorbed through the digestive system and detected in the systemic circulation of humans and/or rats [13–17].

### 2.2. Animals

Male Sprague-Dawley rats were purchased from Japan SLC (Shizuoka, Japan) and used at 7 weeks old. The animals were allowed free access to water and standard laboratory food and housed at a temperature of 23 ± 3°C, relative humidity of 55 ± 20%, and a 12 h light : 12 h dark cycle, with lights on from 07:00 to 19:00 h daily. All experimental procedures were performed according to the “Guidelines for the Care and Use of Laboratory Animals” of Tsumura & Co. Ethical approval of the experimental procedures was obtained from the Laboratory Animal Committee of Tsumura & Co.

### 2.3. *P. acnes*-Induced Acute Dermatitis and Measurement of Ear Thickness

The* P. acnes* strain ATCC6919 was purchased from Microbiologics Inc. (St. Cloud, MN) and cultured in GAM broth (Nissui Pharmaceutical Co., Tokyo, Japan) in an anaerobic atmosphere using a GasPak system (Mitsubishi Gas Chemical Co., Tokyo, Japan). To prepare the* P. acnes* suspension for dermatitis experiments in rat ears,* P. acnes* was harvested, washed with phosphate-buffered saline (PBS), and then centrifuged at 10,000 g for 10 min. After washing twice with PBS,* P. acnes* was heat-killed at 95°C for 5 min, freeze-dried, and then stored at −80°C until use.

*P. acnes*-induced dermatitis in rats was induced using the procedure described by De Young et al. with a minor modification [18]. Heat-killed* P. acnes* (0.14 mg in 50 *μ*L saline) or saline alone was intradermally injected into the ventral side of both ears using a microsyringe (Hamilton Co., Reno, NV) under isoflurane anesthesia. JHT was given orally to rats at 0.1, 0.5, 1, or 2 g/kg in distilled water, 1 h before, and 6, 24 h, 2, 3, 4, 5, and 6 d after the bacterial injection. Prednisolone (PDN) (Shionogi Pharmaceutical Co., Osaka, Japan) was given orally to rats at a dose of 10 mg/kg in distilled water. The ear thickness was measured using a dial thickness gauge micrometer (Ozaki MFG Co., Tokyo, Japan), at 0, 2, 4, 6, 24 h, 2, 3, 4, 5, 6, and 7 d after the bacterial injection. All data on the increase in ear thickness were expressed as a percentage of the previous value in each individual rat.

### 2.4. Histological Evaluation of Inflamed Ears

Some rats were sacrificed by exsanguination under anesthesia 2, 24 h, and 7 d after the bacterial injection. The inflamed ears were excised and immediately fixed with 4% paraformaldehyde at 4°C for histological examinations or frozen in liquid nitrogen for immunological examinations. For histological observation, paraffin-embedded ears were cut into 4 *μ*m cross-sections through ear including the central cartilage. The cross-sections were stained with hematoxylin and eosin (HE) and then visualized by a microscope for the evaluation of cell-mediated inflammatory response. For immunofluorescent (IF) analysis, sections were stained with 1 *μ*g/mL of PE-labeled antimacrophage (clone: HIS36, BD Biosciences, San Diego, CA) and then mounted using the ProLong Gold Antifade Mountant with DAPI (Life Technologies, Grand Island, NY). This antibody reacts with an ED2-like antigen and binds to tissue macrophages but not monocytes. All images were taken using Biorevo BZ-9000 fluorescence microscope and BZ-II software (Keyence, Osaka, Japan). Accumulation of macrophages in the inflamed ears was evaluated as area of PE-positive cells per unit area inside or outside the abscess.

### 2.5. Measurement of Chemokines in Inflamed Ears

The ears obtained at 2 and 24 h after the injection of bacteria were applied to chemokine measurement. In brief, disks of 8.0 mm diameter were punched out and weighed. Protein extracts were obtained by homogenization of ear segments in 0.5 mL of cold PBS supplemented with a cocktail of proteinase inhibitors (Complete, Roche Applied Science, Indianapolis, IN), under the manufacturer's instructions. Samples were centrifuged at 16,000 g for 5 min at 4°C and stored at −80°C until use. MCP-1, CINC-1, and MPO were quantified using ELISA kits according to the manufacturer's instructions (R&D Biosystems, Minneapolis, MN). Total protein was measured by the Lowry assay (Bio-Rad, Hercules, CA) using bovine serum albumin as a standard. Cytokine determinations were compensated and indicated as relative amounts.

### 2.6. Activation-Marker Antigen Assay

Cells of the human monocytic cell line THP-1 (ATCC, Manassas, VA) were grown in RPMI 1640 medium supplemented with 10% heat-inactivated fetal bovine serum, 2 mmol/L L-glutamine, 100 U/mL penicillin, 100 *μ*g/mL streptomycin, and 10 mmol/L HEPES. Cells were seeded in 96-well culture plates at 2 × 10^4^ cells/well and cultured with the test compound (30 *μ*mol/L) in the presence or absence of 10 ng/mL human IFN-*γ* (PeproTech, Rocky Hill, NJ). Two days after incubation in a CO_2_-gas incubator, cells were harvested and analyzed for cell-surface expression of macrophage activation markers. That is, cells were incubated on ice for 20 min with FITC-labeled anti-human CD86 (clone: 2331, BD Biosciences) or PE-labeled anti-human CD192 (clone: K036C2, Biolegend, San Diego, CA), washed, treated for 15 min at 4°C with phosphate buffer containing 4% paraformaldehyde (pH 7.4), and analyzed using an FACScalibur flow cytometer and CellQuest Pro software (BD Biosciences). Levels of expression of CD86 and CD192 were indicated as mean fluorescence intensity (MFI) subtracted with that of nontreated cells. FITC- or PE-labeled isotype-matched control antibodies (BD Biosciences) were used in this study, confirming that the antibodies to CD86 or CD192 showed specific binding.

### 2.7. Phagocytosis Assay

THP-1 cells were seeded in 96-well culture plates at 1 × 10^4^ cells/well and precultured for 3 d in the presence or absence of 10 ng/mL human IFN-*γ*. Test compounds were added to cultures at 30 *μ*mol/L, and Fluoresbrite Yellow Green (YG) Carboxylate Microspheres (2.0 *μ*m, Polysciences, Eppelheim, Germany) were added at a final concentration of 6 × 10^6^ particles/70 *μ*L/well and gently mixed. Two hours after incubation in 5% CO_2_-gas, cells were harvested, washed, and treated for 15 min at 4°C with phosphate buffer containing 4% paraformaldehyde (pH 7.4). For measurement of phagocytosis, FITC-positive cells were determined by an FACScalibur flow cytometer and CellQuest Pro software. Activity was indicated as frequency of FITC-positive cells and MFI of whole cells.

### 2.8. Statistical Analysis

All values are expressed as the mean ± SEM. Statistical significance was evaluated by one-way analysis of variance (ANOVA) or two-way repeated measures ANOVA, followed by the post hoc Bonferroni's or Dunnett's multiple comparisons or unpaired Student's *t*-test. A probability of less than 0.05 was considered significant.

## 3. Results

### 3.1. Antidermatitis Effect of JHT

**Figure 1:**
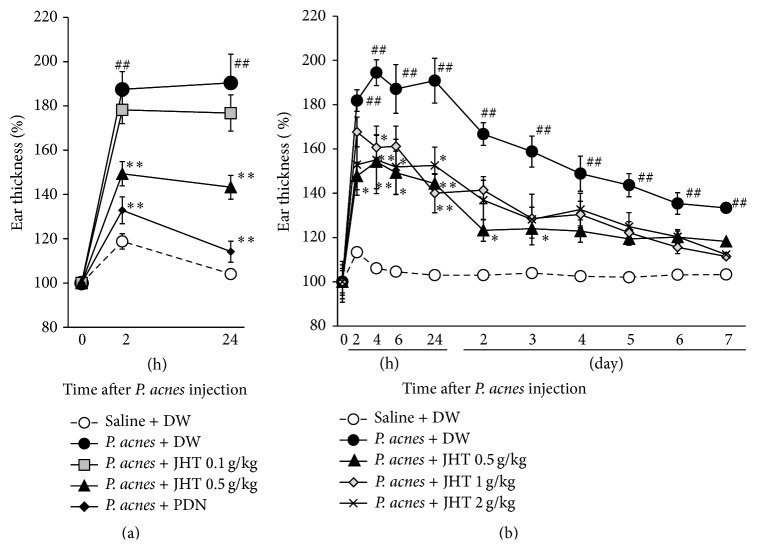
Suppression of* P. acnes*-induced dermatitis in rats by jumihaidokuto administration. Dermatitis was induced by intradermal injection of heat-killed* P. acnes* bacteria suspended in saline (0.14 mg/50 *μ*L/ear) into the pinna. The thickness of the ears was measured at 0, 2, 4, 6, and 24 h and 2, 3, 4, 5, 6, and 7 d after the injection. Jumihaidokuto (JHT) suspended in distilled water (DW) was given orally to rats at doses of 0.1, 0.5, 1, or 2 g/10 mL/kg, at 1 h before 6, 24 h, 2, 3, 4, 5, and 6 d after the injection. Prednisolone (PDN, 10 mg/10 mL/kg) was used as a reference drug. Data represent the relative ear thickness, which was standardized against the previous values before the injection. *N* = 10 (a) or 6-7 (b). ^##^ *P* < 0.01 versus saline + DW group, ^*∗*, *∗∗*^ *P* < 0.05, 0.01 significant versus* P. acne* + DW group (two-way repeated measures ANOVA + Bonferroni test).

We first investigated whether JHT ameliorated* P. acnes*-induced dermatitis in rat ears. Body weights of rats treated with JHT (0.1 to 2 g/kg) were unchanged compared with those of vehicle-given rats throughout the experiments, and no abnormality was observed in these groups (data not shown). Rats subjected to intradermal injection of* P. acnes* and given orally distilled water (control) developed rapid ear swelling with cutaneous erythema. At 2 h after the injection, the ear thickness of control rats had increased to 190% of that prior to injection, and the swelling lasted 24 h (Figure 1(a)). Rats treated with 0.5 g/kg JHT exhibited 149% and 143% increases in ear thickness at 2 and 24 h, respectively, showing an increase about half of control rats, while 0.1 g/kg JHT was not effective. PDN completely inhibited the increase of ear thickness. To examine effects of JHT at higher doses and longer duration, JHT was given at doses of 0.5, 1, or 2 g/kg and observed until 7 d after the* P. acnes* injection. As shown in Figure 1(b), doses of 0.5 to 2 g/kg JHT suppressed the ear swelling due to* P. acnes* injection, though the efficacy was not dose-dependent.

### 3.2. Enhancement of Macrophage Infiltration by JHT

**Figure 2:**
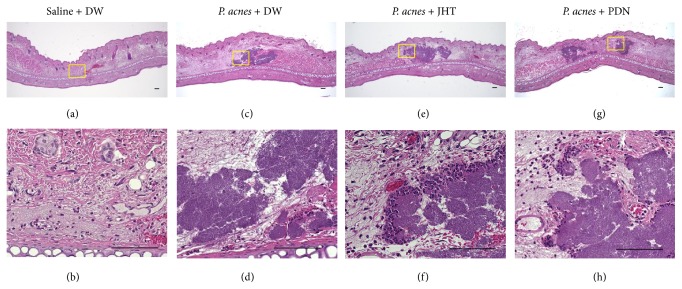
HE staining of pinna 2 h after* P. acnes* injection. The pinnae were excised at 2 h after the* P. acnes* injection and evaluated histologically by hematoxylin and eosin (HE) staining. Representative images are shown for saline + DW ((a) and (b)),* P. acnes* + DW ((c) and (d)),* P. acnes* + 0.5 g/kg jumihaidokuto (JHT) ((e) and (f)), and* P. acnes* + 10 mg/kg prednisolone (PDN) ((g) and (h)). Yellow boxes indicate the sites of each magnification. Scale bars: 100 *μ*m.

**Figure 3:**
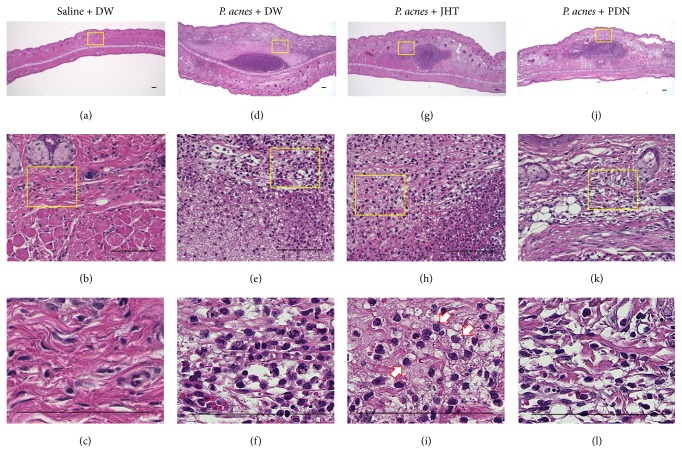
HE staining of pinna 24 h after* P. acnes* injection. The pinnae were excised at 24 h after the* P. acnes* injection and evaluated histologically by hematoxylin and eosin (HE) staining. Representative images of low-power (upper panels) and high-power (middle and lower panels) fields in pinna sections are shown for saline + DW ((a), (b), and (c)),* P. acnes* + DW ((d), (e), and (f)),* P. acnes* + 0.5 g/kg jumihaidokuto (JHT) ((g), (h), and (i)), and* P. acnes* + 10 mg/kg prednisolone (PDN) ((j), (k), and (l)). Yellow boxes indicate the sites of each magnification, and arrows indicate macrophage-like cells. Scale bars: 100 *μ*m.

**Figure 4:**
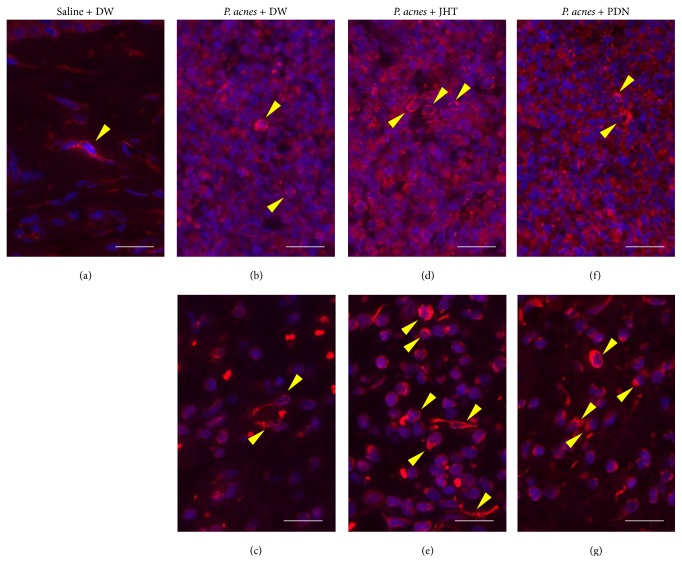
Immunofluorescent staining of macrophages 24 h after* P. acnes* injection. Pinnae sectioned at 4 *μ*m were incubated with a PE-labeled anti-rat macrophage subset monoclonal antibody (clone: HIS36) and then mounted with antifade reagent with DAPI. Representative images merged with DAPI are shown for saline + DW (a),* P. acnes* + DW ((b) and (c)),* P. acnes* + 0.5 g/kg jumihaidokuto (JHT) ((d) and (e)), and* P. acnes* + 10 mg/kg prednisolone (PDN) ((f) and (g)). Panels (b), (d), and (f) are images of the inside of abscesses, and panels (c), (e), and (g) are the outside of abscess. Arrowheads indicate macrophages, which stained double positively with PE and DAPI. PE-positive and DAPI-negative cells found in the images are thought to be red blood cells and regarded as false positives for analysis of macrophages. Scale bars: 20 *μ*m.

**Figure 5:**
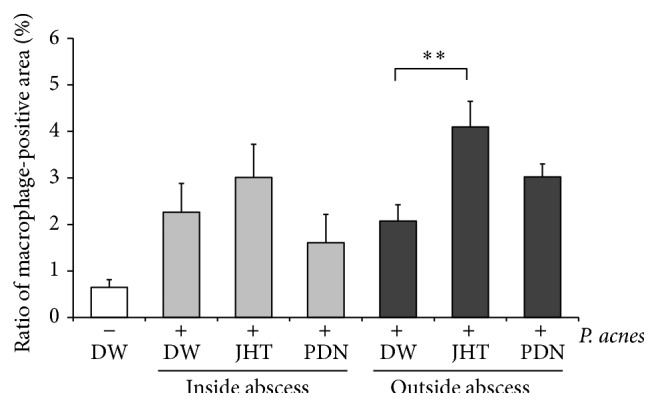
Jumihaidokuto promoted macrophage accumulation in inflamed ears. Macrophages were stained by the procedures described in Figure 4. Accumulation of macrophages in the inflamed ears was evaluated relative to areas of PE-positive cells per unit area inside or outside the abscess using Biorevo BZ-9000 microscope and BZ-II software (Keyence, Osaka, Japan). *N* = 5. ^*∗∗*^ *P* < 0.01 (Dunnett's test).

Histological examination of inflamed ears in control rats showed infiltration of inflammatory cells at 2 h, 24 h, and 7 d, with evident abscess at 24 h. Representative images at 2 and 24 h are shown in Figures 2 and 3, respectively. In images of HE stains showing* P. acnes* deposition and infiltrated cells at 2 h after* P. acnes* injection, there was no obvious difference among three groups of rats given 0.5 g/kg JHT, PDN, and distilled water (Figure 2). However, in the magnified images, we observed that more epithelioid macrophages had infiltrated the whole inflamed area at 24 h in rats given JHT than in PDN or control rats (Figure 3). IF stain using an anti-rat macrophage antibody showed that macrophages had accumulated around the abscess at 24 h (Figure 4). The accumulation of macrophages in each group was semiquantified and is shown in Figure 5. These results revealed that rats given JHT showed significantly more macrophage accumulation in the dermatitis induced by* P. acnes* than control rats. Rats given PDN showed less macrophage infiltration than rats given JHT, though the differences were not statistically significant. We also examined the amounts of MPO, a neutrophil marker, in the inflamed ears and observed similar increases in neutrophil infiltration in rats given JHT and control rats at 2 and 24 h but less infiltration in rats given PDN (data not shown). These suggested that JHT enhances macrophage infiltration in* P. acnes*-induced dermatitis.

**Figure 6:**
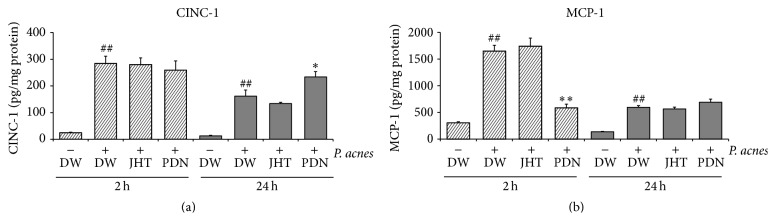
Jumihaidokuto had no effect on chemokine concentrations in inflamed ears. The pinnae were collected at 2 and 24 h after injection of* P. acnes*. Each sample was homogenized in cold PBS supplemented with proteinase inhibitors and centrifuged. CINC-1 (a) and MCP-1 (b) concentrations in the supernatants were determined by ELISA. Jumihaidokuto (JHT, 0.5 g/kg), prednisolone (PDN, 10 mg/kg), or distilled water (DW) was administered orally 1 h before and 6 h after bacterial injection. *N* = 10 (2 h), 4-5 (24 h). ^##^ *P* < 0.01 significant versus saline + DW group (Student's *t*-test), ^*∗*, *∗∗*^ *P* < 0.05 and 0.01, respectively, and significant versus* P. acnes* + DW group (Dunnett's test).

To elucidate the mechanisms of the increased macrophage accumulation in rats given JHT, we examined the macrophage chemokines in inflamed ears. We observed increases in two chemokines, MCP-1 and CINC-1 (a homolog to human IL-8), in the inflamed ears at both 2 and 24 h after* P. acnes* injection. JHT did not augment the induction, while PDN suppressed MCP-1 induction at 2 h (Figure 6).

### 3.3. Investigation of JHT-Related Compounds Modulating Macrophage Functions

The results of the induction of dermatitis in the animal model by* P. acnes* allowed us speculate that JHT modulates the biological functions of macrophages, and we therefore performed cell-based assays using the human monocyte cell line THP-1. In this study, we examined six compounds, which were all reported to be quantifiable in human and/or animal blood. They were 18*β*-glycyrrhetinic acid, liquiritin, liquiritigenin, isoliquiritin, 4-*O*-methylgallic acid, and cimifugin. We performed blood pharmacokinetics by MS spectrometry in rats given JHT and confirmed that the six compounds were detected in the peripheral blood of rats (data not shown).

We examined the effects of the six compounds on IFN-*γ*-stimulated THP-1 cells by measuring a differentiation marker, CD86, for type-1 macrophages (M1). As shown in Table 1, liquiritin, isoliquiritin, liquiritigenin, and cimifugin significantly enhanced CD86 expression in IFN-*γ*-treated THP-1 cells compared with IFN-*γ*-alone control. They also enhanced expression of CD192, a receptor of MCP-1, which was increased in the inflamed skin in* P. acnes* dermatitis. Finally, we performed phagocytosis assays. THP-1 cells treated with liquiritin or liquiritigenin in the presence of IFN-*γ* exerted higher phagocytic activity than the IFN-*γ*-treated alone control (Table 1). These findings suggest that JHT enhanced differentiation of monocytes towards M1 macrophages, which can be efficiently recruited to inflamed skin by CD192 and MCP-1.

## 4. Discussion

To the best of our knowledge, this is the first report showing that JHT modifies macrophage functions. JHT suppressed increases in ear thickness induced by intradermal injection of* P. acnes* as well as the immunosuppressive agent PDN did. However, histological examination at 24 h after the* P. acnes* injection showed a difference between the two drugs. Interestingly, JHT had dramatically accelerated migration of tissue macrophages in the inflamed ears at 24 h, in contrast to suppression of inflammatory cell infiltration by PDN. Moreover, JHT enhanced monocyte differentiation toward M1 and enhanced MCP-1 receptor expression as well as phagocytic activity. These suggested that JHT activates macrophages and enhances their recruitment to the skin with the dermatitis. Considering the inhibitory effect of JHT on ear thickness at 24 h and thereafter, the enhancement of macrophage accumulation was thought to attenuate or accelerate the resolution of the dermatitis. To examine the pathophysiological significance of macrophage accumulation in the inflamed ears, a Gram-stain analysis was performed preliminarily. That is, the number of Gram-positive depositions of the inoculated* P. acnes* components per unit area outside the abscess was calculated, resulting in a negative correlation between macrophage accumulation and stained bacteria (data not shown). Although this needs further study, it is possible that JHT promotes clearance of the bacteria.

JHT enhanced macrophage accumulation* in vivo* and differentiation to type-1 macrophages* in vitro*, while JHT inhibited inflammation. These results are confusing because activation of macrophages, especially type-1 macrophages, is known to evoke inflammatory responses. Therefore, we examined the balance between type-1 and type-2 macrophages in the assay shown in Table 1 to assess activation markers. As a result, expression of the type-2 marker CD206 (mannose receptor) was significantly increased, as well as the type-1 markers CD86 and CD192, by the active constituents of JHT (data not shown). Namely, JHT is supposed to activate macrophages nonselectively, not to develop an extreme polarization to type-1 macrophages.

One of the hallmarks of macrophages is their remarkable plasticity. Type-1 and type-2 macrophages are theoretically categorized by their biological functions. Macrophage polarization can be affected by concurrent stimulation of several different signaling pathways. According to recent reports, the identification of a “switch” from a type-1 to a type-2 phenotype provides a novel paradigm for a relationship between macrophage polarization and the inflammation process [19–21]. This indicates that type-1 macrophages and/or monocytes migrated to the inflamed site, can be converted to type-2 macrophages by exposure of a modifier-like adenosine and pyrophosphates, and substantially work as anti-inflammatory cells, for example, releasing the regulatory cytokine IL-10. It is known that a proper inflammation is essential for normal wound healing, especially in infectious inflammation [19, 22]. JHT may globally promote the physiological defense system, that is, stimulating a series of actions from proper inflammation to its termination and prompt wound healing.

The content of MCP-1 in the inflamed ears 2 h after the bacteria injection was not affected by oral administration of JHT. MCP-1 and other inflammation-related cytokines and chemokines like IL-1*α*, IL-1*β*, IL-10, TNF-*α*, and CINC-1 in the inflamed ears at 2 and/or 24 h were the same in rats given JHT as those in control rats (data not shown). These results allowed us to hypothesize that the effect of JHT on accumulation of macrophages resulted from modification of monocytes in the systemic blood and/or digestive tract including the liver and intestines. The cell-based assay revealed that liquiritin, liquiritigenin, isoliquiritin, and cimifugin increased expression of MCP-1 receptor and CD192 (CCR2), as well as activation-marker CD86. We and other investigators have demonstrated that these constituents of JHT were absorbed rapidly into the peripheral blood [13–15]. Therefore, the enhancement of macrophage accumulation in the inflamed ears may be partially due to enhanced MCP-1 receptor expression in circulating monocytes/macrophages due to active JHT constituents before and during migration toward the site of the dermatitis. It is also noteworthy that liquiritigenin and isoliquiritin enhanced phagocytic activity of the human monocyte cell line THP-1. Although the present study did not show any effects on neutrophils* in vivo*, a previous report implied that JHT increased chemotaxis and phagocytosis of human neutrophils in* in vitro* assays [9]. Thus, these compounds may exert adjuvant effects by activating macrophage functions and regulating sterile inflammation.

*Glycyrrhiza* radix, one of crude components of JHT, is well known to exert various pharmacological properties including antioxidative, anti-inflammatory, antimicrobial, hepatoprotective, and immunomodulatory activities [15, 23]. The main flavonoids of* Glycyrrhiza* radix are liquiritin (and its apioside), liquiritigenin, isoliquiritin (and its apioside), isoliquiritigenin, formononetin, and coumestrol, which are generated by a series of metabolisms [15, 24]. Several investigators reported that these flavonoids ameliorate inflammation [25–27]. Liquiritigenin inhibited formation of paw edema induced by carrageenan in rats [28] and IgE-dependent acute dermatitis [29]. We reported quantification of 20 polyphenols including gallic acid in the* Quercus acutissima* cortex, one of the crude components of JHT [30]. 4-*O*-Methylgallic acid, a main metabolite of gallic acid, appears in blood and urine after oral administration of gallic acid or black tea rich in gallotannin and gallic acid [16, 17]. 4-*O*-Methylgallic acid shows antiangiogenic and anti-inflammatory effects* in vitro* [31, 32], implying a potential to treat rosacea. Cimifugin is one of the constituents* Saposhnikovia divaricatae*. In pharmacokinetic studies, oral administration of a cimifugin-containing crude drug to rats resulted in a high concentration of cimifugin in the blood [13, 14]. For instance, cimifugin was measured at 881–1510 ng/mL in the blood of rats given a traditional Chinese medicine (Yu Ping Feng San) [33]. Cimifugin suppressed nitric oxide production by murine macrophages and exhibited 1,1-diphenyl-2-picrylhydrazyl free-radical scavenging activity in a cell-free bioassay system [34].

Acne vulgaris is an inflammatory disease of the sebaceous glands and pilosebaceous units in the skin [3]. Environmental stimuli such as ultraviolet light and free fatty acids produced by* P. acnes* living in sebaceous glands are typical triggers of inflammation in early stages of acne vulgaris. Excessive inflammation including overactivation of migrated neutrophils leads to rupture of sebaceous follicle walls, resulting in severe inflammation and formation of papules and pustules by direct interaction between* P. acnes* and host cells, especially neutrophils and macrophages. The present study revealed that rats treated by JHT had accelerated macrophage accumulation in the inflamed ears induced by* P. acnes*, but not by PDN. Given that these events are closely related to a model of acne vulgaris, the anti-inflammatory mechanisms of JHT would be important findings for clinical use. Further studies of JHT may help to understand the pathophysiological features of regulatory macrophage and develop its therapeutic application.

## 5. Conclusions

JHT suppressed dermatitis in a rat model of acne vulgaris induced by intradermal injection of* P. acnes*. The results of HE and IF stains revealed that JHT promoted macrophage accumulation in the inflamed ears. Two JHT constituents, liquiritigenin and isoliquiritin, activated human monocytes by increasing CD86 and CD192 expression and phagocytosis. JHT may have an anti-inflammatory function with an adjuvant effect of enhancing macrophage functions and may be beneficial in a therapeutic strategy for skin inflammation disorders including acne.

## Supplementary Material

Chemical structures of liquiritin, liquiritigenin, isoliquiritin, cimifugin, 18β-glycyrrhetinic acid (main metabolite of glyccyrrhizin), and 4-O-methylgallic acid (the main metabolite of gallic acid), are shown.

## Figures and Tables

**Table 1:** Effects of jumihaidokuto-related compounds on macrophage functions.

Test compound	IFN-*γ* (10 ng/mL)	Expression of activation marker		Phagocytosis	
		CD86	CD192	% of FITC^+^ cells	MFI
—	−	0.08 ± 0.06	0.19 ± 0.16	14.43 ± 1.08	3.16 ± 0.17
—	+	1.70 ± 0.07	1.57 ± 0.05	20.86 ± 1.05	4.76 ± 0.28
Liquiritin	+	3.28 ± 0.10^*∗∗*^	3.76 ± 0.05^*∗∗*^	24.67 ± 1.24	6.07 ± 0.41
Liquiritigenin	+	3.47 ± 0.12^*∗∗*^	3.80 ± 0.19^*∗∗*^	31.01 ± 3.06^*∗∗*^	7.88 ± 0.93^*∗∗*^
Isoliquiritin	+	5.27 ± 0.17^*∗∗*^	4.21 ± 0.15^*∗∗*^	42.42 ± 1.24^*∗∗*^	12.94 ± 0.71^*∗∗*^
18*β*-Glycyrrhetinic acid	+	1.84 ± 0.13	2.24 ± 0.12	18.00 ± 1.45	4.18 ± 0.31
Cimifugin	+	3.82 ± 0.43^*∗∗*^	4.84 ± 0.65^*∗∗*^	19.05 ± 0.59	4.28 ± 0.10
4-*O*-Methylgallic acid	+	1.18 ± 0.10	1.18 ± 0.12	18.91 ± 1.36	4.27 ± 0.31

Human monocytic THP-1 cells were seeded in 96-well culture plates at 1 or 2 × 10^4^ cells/well and cultured with test samples (30 *μ*mol/L) in the presence or absence of 10 ng/mL human IFN-*γ*. To examine the activation markers, cells were harvested 2 d later and stained with a fluorescent-labeled antibody specific to CD86 or CD192. Their expressions in cells were determined by flow cytometry. Data are shown as mean fluorescence intensity (MFI) subtracted with MFI of naive cells (CD86: 7.29, CD192: 11.60). In the study of phagocytosis, FITC-microspheres were added at 6 × 10^6^ beads/well after the preculture for 3 d and cultured for an additional 2 h. Cells were harvested, and the FITC-positive cells were measured. *N* = 3. ^*∗∗*^ *P* < 0.01 versus IFN-*γ* alone control (Dunnett's test).
