# Supplementary material for: Suppression of Propionibacterium acnes-Induced Dermatitis by a Traditional Japanese Medicine, Jumihaidokuto, Modifying Macrophage Functions
Source: Evid Based Complement Alternat Med. 2015 Oct 1;2015:439258. doi: 10.1155/2015/439258 (PMC4606168; doi:10.1155/2015/439258)
Supplement: Supplementary file 1 — Chemical structures of liquiritin, liquiritigenin, isoliquiritin, cimifugin, 18β-glycyrrhetinic acid (main metabolite of glyccyrrhizin), and 4-O-methylgallic acid (the main metabolite of gallic acid), are shown. [file 439258.f1.pdf]

Supplemental Materials

Supplementary Fig. S1. Chemical structures of jumihaidokuto-related compounds evaluated in this study

liquiritin
Glycyrrhizae radix

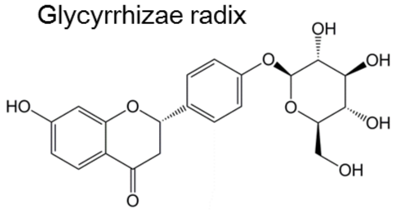

liquiritigenin
Glycyrrhizae radix

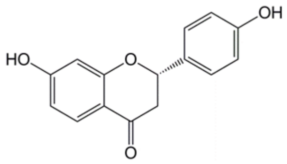

Isoliquiritin
Glycyrrhizae radix

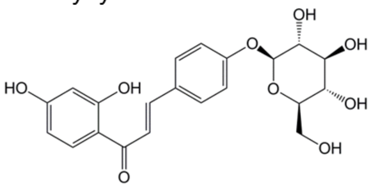

18β-glycyrrhetinic acid
Glycyrrhizae radix

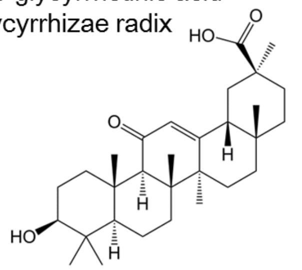

cimifugin
Saposhnikoviae radix

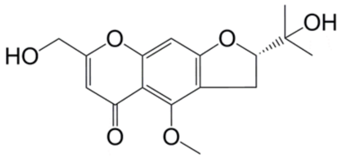

4-*O*-methylgallic acid
Quercus cortex

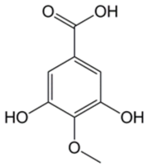

Supplementary Fig.S1.
